# Fast Holocene slip and localized strain along the Liquiñe-Ofqui strike-slip fault system, Chile

**DOI:** 10.1038/s41598-021-85036-5

**Published:** 2021-03-16

**Authors:** Luis Astudillo-Sotomayor, Julius Jara-Muñoz, Daniel Melnick, Joaquín Cortés-Aranda, Andrés Tassara, Manfred R. Strecker

**Affiliations:** 1 Millennium Nucleus the Seismic Cycle Along Subduction Zones, Valdivia, Chile; 2 Departamento de Ciencias de la Tierra, Universidad de Concepción, Concepción, Chile; 3 Institute of Geosciences, University of Potsdam, Potsdam, Germany; 4 Instituto de Ciencias de la Tierra, TAQUACh, Universidad Austral de Chile, Valdivia, Chile

**Keywords:** Geodynamics, Geomorphology, Tectonics

## Abstract

In active tectonic settings dominated by strike-slip kinematics, slip partitioning across subparallel faults is a common feature; therefore, assessing the degree of partitioning and strain localization is paramount for seismic hazard assessments. Here, we estimate a slip rate of 18.8 ± 2.0 mm/year over the past 9.0 ± 0.1 ka for a single strand of the Liquiñe-Ofqui Fault System, which straddles the Main Cordillera in Southern Chile. This Holocene rate accounts for ~ 82% of the trench-parallel component of oblique plate convergence and is similar to million-year estimates integrated over the entire fault system. Our results imply that strain localizes on a single fault at millennial time scale but over longer time scales strain localization is not sustained. The fast millennial slip rate in the absence of historical Mw > 6.5 earthquakes along the Liquiñe-Ofqui Fault System implies either a component of aseismic slip or Mw ~ 7 earthquakes involving multi-trace ruptures and > 150-year repeat times. Our results have implications for the understanding of strike-slip fault system dynamics within volcanic arcs and seismic hazard assessments.

## Key Points

For the first time we estimate a Holocene slip rate for a single fault strand of the Liquiñe-Ofqui Fault System, a major strike-slip fault in Chile. This fault slips at 18.8 mm/yr and accommodates ~82% of the trench-parallel component of oblique plate convergence. The lack of historical earthquakes along the Liquiñe Fault suggests either a component of aseismic slip or large sporadic Mw~7 earthquakes.

## Introduction

**Figure 1:**
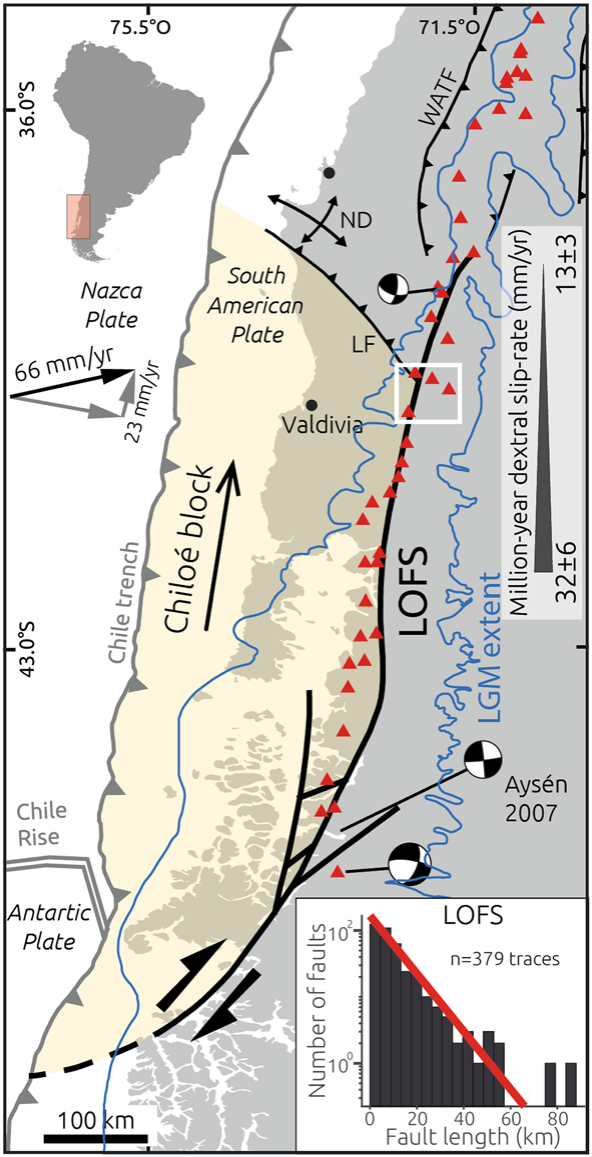
Seismotectonic setting map of the Patagonian Andes. Focal mechanisms of mayor earthquakes along the Liquiñe-Ofqui Fault System (LOFS) from the CMT Catalogue. Black and grey arrows show Nazca-South America plate convergence vector^39^ and decomposed margin-normal and margin-parallel components. Simplified regional faults from Ref.^18^. LF-Lanalhue Fault; ND-Nahuelbuta Dome. Inset shows the distribution of fault lengths for all the LOFS traces from the CHAF database^20^.

In most subduction zones, the direction of plate convergence is oblique to the trench^1^; this oblique convergence may be accommodated and partitioned either along distinct upper-plate faults or across broad zones of diffuse deformation^2^. When slip associated with oblique plate convergence is completely partitioned, the trench-normal component of motion is accounted for by megathrust earthquakes, while the trench-parallel component is accommodated by continental strike-slip fault systems^3,4^. Such continental strike-slip fault systems may localize slip along thermally-weakened volcanic arcs such as in Sumatra, the Philippines, Central America, Japan and south-central Chile^3,5–8^. In such tectonic settings, fundamental steps in seismic hazard assessments involve quantifying the degree and the spatiotemporal patterns of slip partitioning, strain localization, and fault-slip behaviour^9^ (stick–slip vs creep). Along the south-central Chile margin (37°–46°S), oblique convergence between the Nazca and South American plates is partitioned between the megathrust and the Liquiñe-Ofqui Fault System (LOFS, Fig. 1, Ref.^8^). The LOFS straddles the active volcanic arc and has generated only a few historical earthquakes that reached up to M_w_ 6.2 (Sielfeld et al.^10^ and references therein). However, the LOFS lacks of well-defined fault segments characterized by unambiguous surface ruptures that may have been associated with prehistoric great earthquakes. In humid southern Chile, such paleoseismic evidence could be subdued due to a combination of efficient erosive processes associated with glaciation and deglaciation that degrade and erase the fault-related morphology. In addition, a dense vegetation cover, such as along the steep western flank of the southern Andean cordillera, and widespread Holocene volcanic deposits mask and limit the number of available outcrops with evidence of recent surface deformation. Alternatively, the apparent lack of evidence for seismogenic surface ruptures might be related to predominant aseismic slip and absence of past great earthquakes with magnitudes larger than in historical times (M_w_ > 6.2). In light of these issues, the seismic potential of the LOFS remains poorly known^11,12^. Slip rates along the LOFS have been estimated at million-year^13^ and decadal^11^ time scales across the entire fault system, but the behaviour of individual fault strands at intermediate time scales is virtually unknown. Only a few faults of probable Holocene age have been mapped to date^12,14–16^, but lack slip-rate determinations and paleoseismic estimates. Here, we focus on a site with exposed neotectonic features and present the first Holocene slip-rate estimate for the LOFS. We integrate our results with shorter and longer-term rates to discuss the implications for strain localization processes, fault-slip behaviour, and seismic hazards.

### Seismotectonic setting of the Southern Andes

The structural setting of the Main Cordillera of the Southern Andean intra-arc zone is dominated by the NNE-striking LOFS, which is limited by the Chile Triple Junction in the south and the Lanalhue Fault in the north (Fig. 1). The LOFS is a ~ 1200-km-long dextral strike-slip fault system that decouples a forearc sliver–the Chiloé Block–from the stable Patagonian foreland^17^. The margin-parallel translation of the Chiloé Block is apparently accommodated at its leading edge in the north by folding and faulting along the Arauco Peninsula and the Nahuelbuta Dome^18^ (Fig. 1). The LOFS is a complex structure including evidence for vertical-axis block rotation, duplexes and horsetail terminations at local and regional scales that are associated with transtensional and transpressional segments^8,13^. The LOFS consists of 1 to 10 subparallel fault strands distributed across the axis of the Andean Main Cordillera with maximum lengths of ~ 60 km (inset in Fig. 1), segmented by Andean transverse structures^19^. The geometry of the LOFS may be appreciated in the Chilean Database of Active Faults^20^. Rosenau et al.^13^ estimated mean shear rates decreasing northward from 32 ± 6 to 13 ± 3 mm/year (Fig. 1), along a ~ 80- to 120-km-wide LOFS during the last 4 Ma. These estimates were based on kinematic modelling using cross-cutting fault geometric relations, and are consistent with vertical-axis rotations from paleomagnetic data^21^.

Wang et al.^22^, based on modelling of GPS velocities, inferred that the Chiloé forearc sliver translates northwards at ~ 6.5 mm/year, which they related to rigid block motion and slip along the LOFS; in turn, Stanton-Yonge et al.^11^ proposed a 1–7 mm/year slip rate for individual LOFS strands based on a boundary element model that relied on GPS velocities of Ref.^22^. However, a limitation of these GPS velocities is that they were based exclusively on campaign measurements, which do not account for seasonal variation due to non-tectonic processes^41^. Seasonal variations with amplitudes of up to 20 mm are evident in the north component (ca. margin parallel) of daily positions estimated from continuous GPS measurements at sites within the Chiloé sliver^23^. Therefore, robust decadal-scale slip rate estimates for the LOFS are yet to be estimated using continuous GPS measurements.

The LOFS is associated with clusters of shallow microseismicity that reach down to 15 km depth^10,24,25^, and with up to M_w_ 6 strike-slip earthquakes recorded during the 1965 Hudson and 1989 Lonquimay volcanic eruptions^26,27^ (Fig. 1). In 2007, a sequence of earthquakes with dextral and normal focal mechanisms occurred along the LOFS at Aysén fjord reaching Mw 6.2 and triggering massive slope failures that caused a local tsunami^12,15,16^. On the bottom of the fjord floor, multibeam bathymetry mapping revealed a 3-km-long submarine surface rupture associated with the 2007 earthquake^16^; these authors also used a record of seismically-induced mass flow events retrieved from a 21-m-long sediment core leading to the estimation of a ~ 2-ka recurrence interval for LOFS-related earthquakes in this region; however, no magnitude could be estimated for those earthquakes. Also at Aysén, Vargas et al.^12^ speculated that M_w_ 6.2–6.5 earthquakes may be expected along most strands of the LOFS and up to M_w_ 7.1 along master faults, but these estimates were not based on paleoseismic observations and lack determinations of recurrence intervals. Kanamori and Rivera^28^ associated a Mw 7.7 slow earthquake that occurred in 1960 close to Aysén to ductile motion at depth below the LOFS based on analysis of strain seismograms and lack of macroseismic effects.

## Results

### Tectonic geomorphology of the Liquiñe site

**Figure 2:**
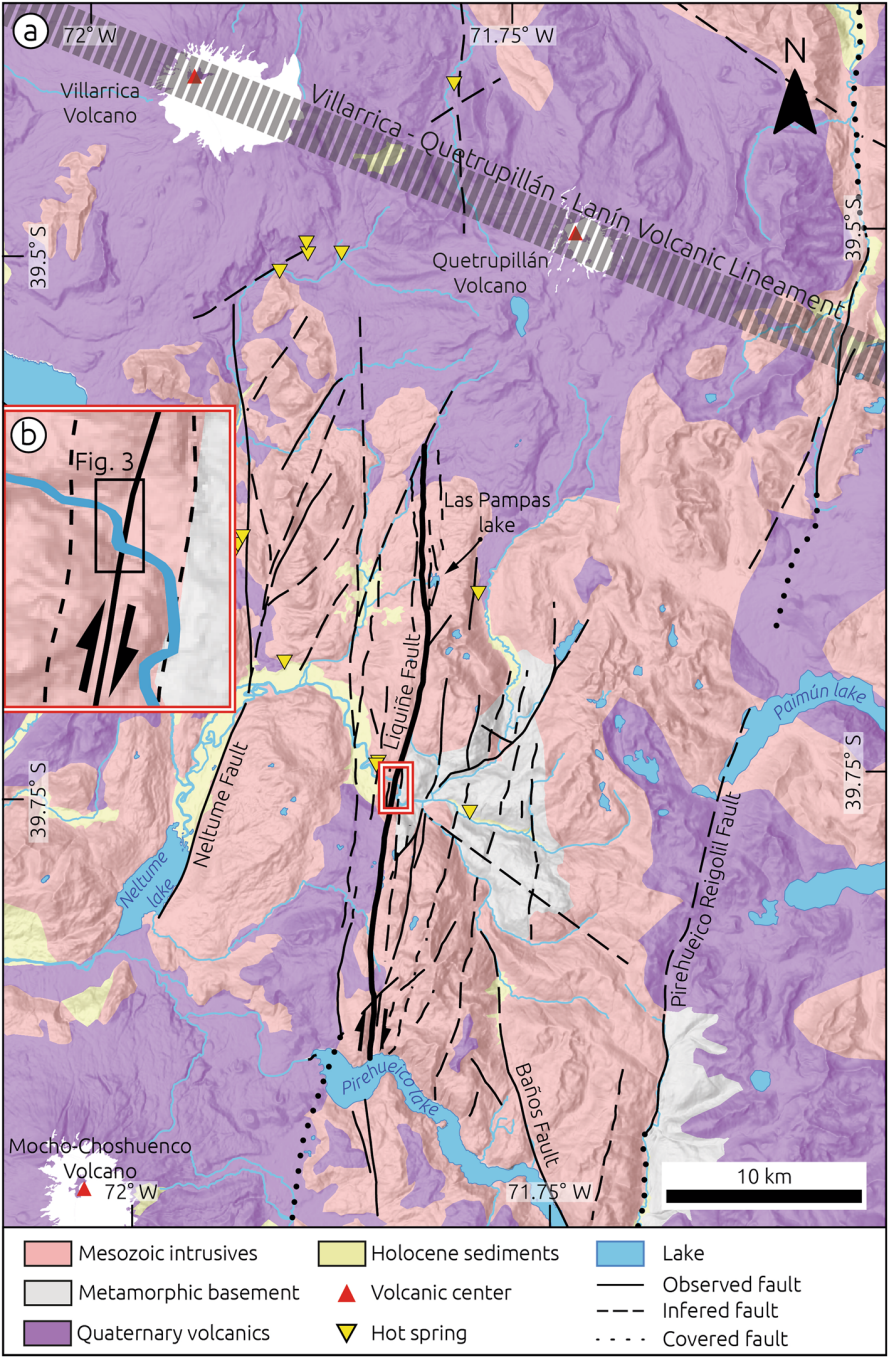
Geologic map of the Liquiñe region (modified from Ref.^40^). Faults from the CHAF database^20^. Thick black line denotes the Liquiñe Fault. (**b**) Detailed view of the Liquiñe Site, note marked deflection of the Liquiñe River.

**Figure 3:**
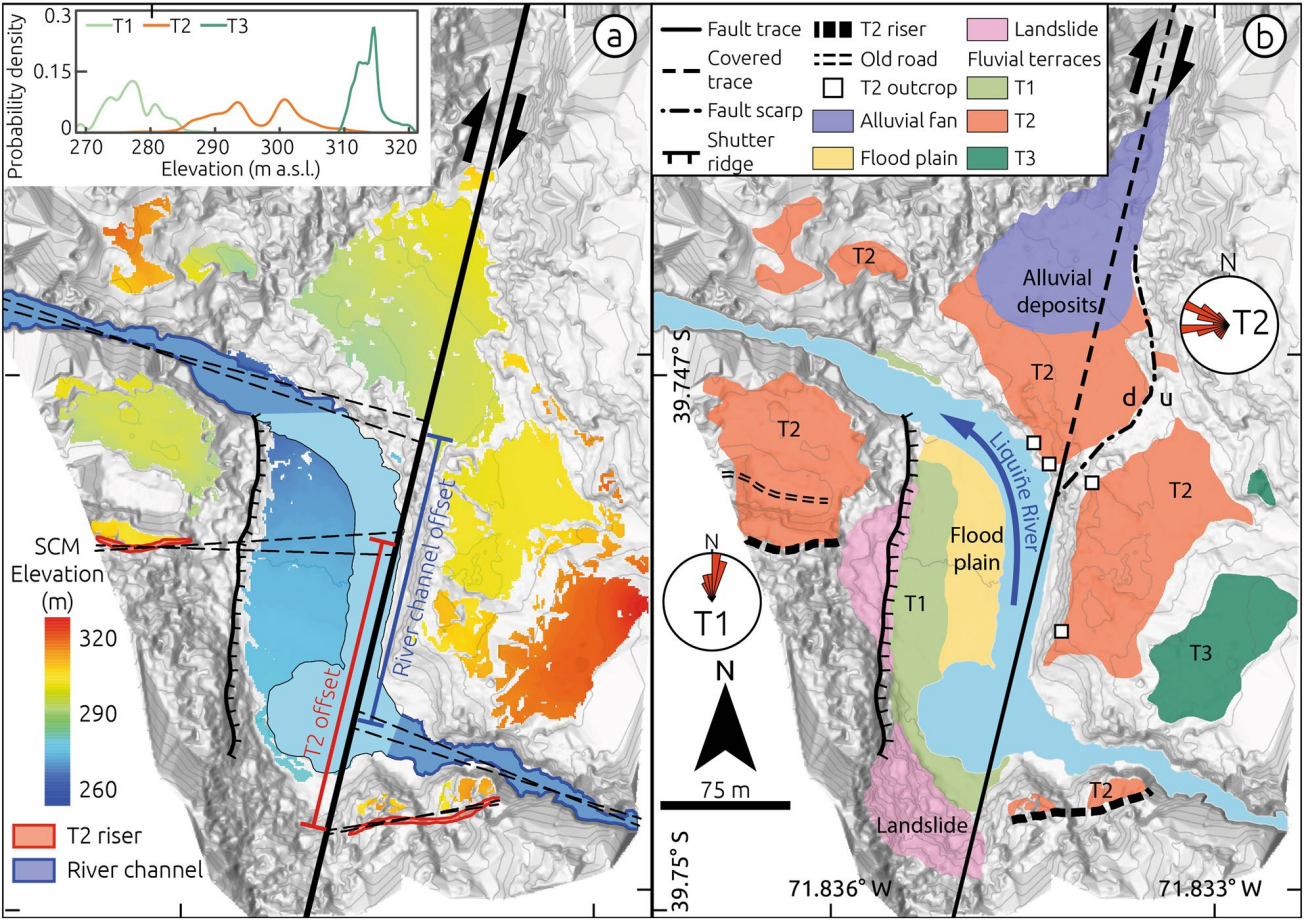
Tectonic geomorphology of the Liquiñe Site. (**a**) Shaded-relief Digital Terrain Model from terrestrial-laser scanner survey overlaid by colour-coded elevation of Surface Classification Model (details in methods and Supplementary Materials). The black thick line shows the Liquiñe Fault trace used to calculate fault slip by projecting two geomorphic markers (T2 and modern river channel). Stippled black lines denote extrapolation piercing lines used to estimate fault offsets. Light grey lines denote 5-m contour intervals. Inset shows elevation distribution of fluvial terraces based on SCM classification. (**b**) Geomorphic map showing distribution of fluvial terraces and structures. Rose plots show paleocurrent directions estimated from clast imbrication measurements at terraces T1 and T2. Histograms of the elevation distribution of T2 at each fault quadrant and topographic profiles across the fault and river supporting the mapping of T2 may be found in the Supplementary Materials (Fig. S4). Sense of vertical displacement across fault scarp affecting T2 denoted by u-up and d-down.

**Figure 4:**
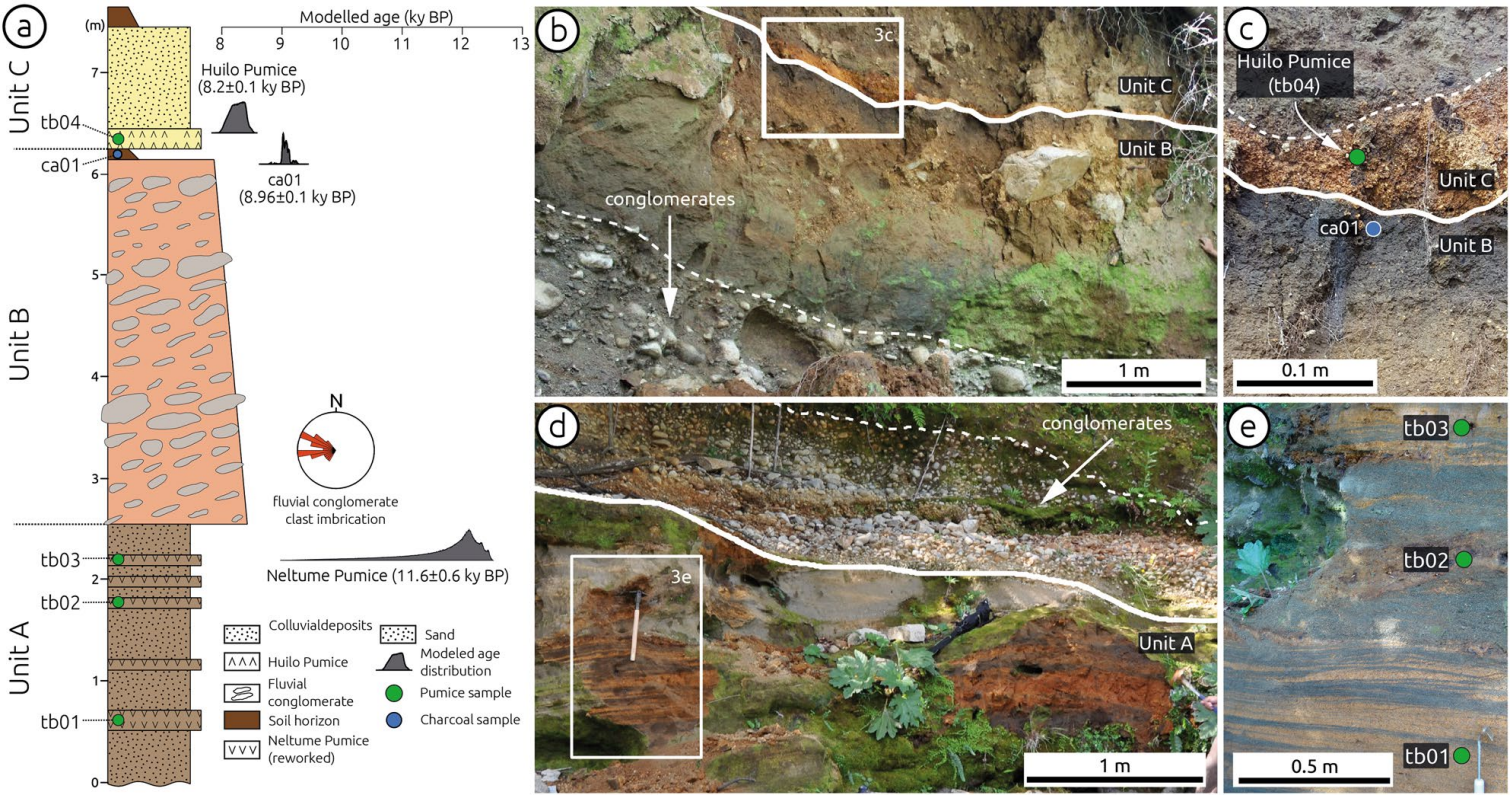
Stratigraphy and geochronology of terrace T2. (**a**) Composite stratigraphic section with units and location of pumice and charcoal samples. Grey probability density plots denote modelled age distributions for stratigraphic markers from OxCal model results (Supplementary Materials, Fig. S7). Rose diagram shows paleocurrent direction estimated from clast imbrication in unit B. Grey curves denote modelled age distributions (see text for details). (**b**) Field view of contact between units B and C. Note upward-fining transitional contact between conglomerate and paleosol (stippled white line), and sharp contact with Unit C (solid white line). (**c**) Detail of contact between units B and C (solid white line). Blue and green circles show sampling locations. Stippled white line marks top of Huilo pumice. (**d**) View of unit the contact between units A and B (thick white line) and of basal conglomerates in unit B. Stippled white line shows dipping layers of the conglomerates. (**e**) Detailed view of reworked Neltume pumice and black volcanic sand in unit A. Green dots show sampling locations. Photos reproduced with consent from copyright holders.

**Figure 5:**
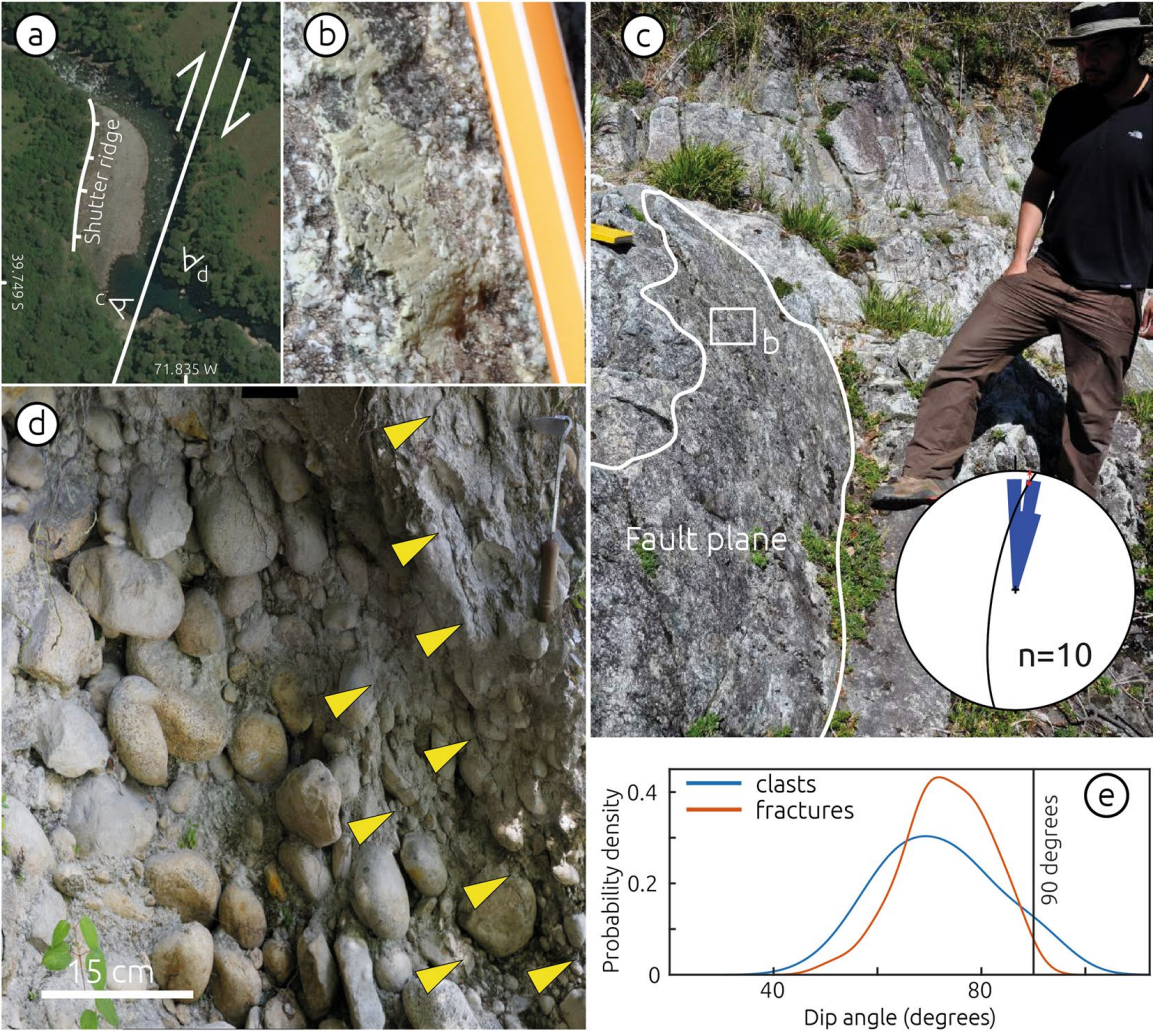
Field evidence of strike-slip faulting at the Liquiñe Site. (**a**) Satellite image showing location of field views in panels (**b**), (**c**) and (**d**), as well as trace of the Liquiñe Fault and extent of shutter ridge. (**b**) Detailed view of subhorizontal slickenside in fault plane shown in (**c**). (**c**) Fault planes affecting granitic bedrock. Rose diagram shows distribution of fault strike measurements (n = 10), constructed using Stereonet v. 11 (http://www.geo.cornell.edu/geology/faculty/RWA/programs/stereonet.html). Black circle and red arrow show stereographic projection of the fault plane and slickenside lineation shown in b, constructed using FaultKin v. 8 (http://www.geo.cornell.edu/geology/faculty/RWA/programs/faultkin.html). (**d**) Field view looking north of shear zone affecting conglomerates of terrace T2. Yellow arrows show trans-granular fractures affecting matrix and clasts. (**e**) Probability density functions of fractures and rotated clasts dips within the shear zone in (**c**). Probability density functions calculated using 64 and 39 measurements of clast long axes and fractures, respectively, from east to west, using MATLAB R2017a (www.mathworks.com). Figure drafted using Inkscape 1.0 (www.inkscape.com). Photos reproduced with consent from copyright holders.

The upper Liquiñe River is associated with a ~ 600-km^2^ catchment characterized by a narrow and steep channel cut into crystalline bedrock in its upper reaches and a meandering system downstream (Fig. 2). The channel follows a series of smooth bends associated with fault strands of the LOFS, and 2 km east of the town of Liquiñe the river is characterized by a pronounced right-angle deflection associated with faulted fluvial terraces as well as folded and faulted sediments (Figs. 3, 4, 5), referred to by us as the Liquiñe Site (inset in Fig. 2b). Here, we identified three fluvial terraces at the Liquiñe Site using field mapping and a Surface Classification Model (SCM) extracted from a Digital Terrain Model (DTM) (Fig. 3a). The SCM is based on a combination of slope and terrain roughness that allows masking flat and smooth areas from the DTM^29^. Details on the acquisition and processing of terrestrial laser scanner data as well as DTM and SCM generation may be found in the Methods section and Supplementary Materials (Text S1, Figs. S1 and S2). The distribution of SCM elevation suggests three distinct terrace surfaces at ~ 270, ~ 300, and ~ 315 m asl (inset in Fig. 3a). Terrace T1 was formed 2 m above the modern floodplain and includes elongated cobbles and pebbles with a well-developed N-oriented imbrication in its central part, which is parallel to the flow direction of the present-day river (T1 in Figs. 3b and S3b). Terraces T2 and T3 are located at 15 and 25–30 m above the riverbed, respectively. T2 is the best-preserved terrace associated with a 15-m-high terrace riser above T1 and smooth surface topography (Figs. 3 and S3c). T2 is locally disrupted by a 5-m-high NE-SW striking fault scarp exposing granitic rocks to the uplifted eastern block (stippled-dotted line in Fig. 3b, field photo in Fig. S3a). The correlation of T2 surfaces across the Liquiñe River was based on the SCM and topographic profiles (Fig. S4).

We subdivided T2 deposits (extent of deposits shown in Fig. 3b) into three units (Fig. 4a): Unit A consists of a 2.5-m-thick, cross-bedded sequence of poorly consolidated sand with lenses of reworked pumice (Fig. 4d, e); Unit B comprises a 3.7-m-thick, matrix-supported and upward-fining fluvial conglomerate of well-rounded clasts associated with an E- to ENE–oriented imbrication (measured sites shown by squares in Fig. 3b, inferred paleocurrent directions shown in Figs. 3b and 4a), which is covered by a well-developed forest paleosol horizon (Fig. 4b, c); and Unit C consists of a 10-cm-thick pumice layer covered by poorly sorted angular colluvial cobbles (Fig. 4b, c). At the top of Unit C the current forest soil horizon has developed. Terrace T2 is partly covered by a younger alluvial fan in its northern part (Fig. 3b).

**Figure 6:**
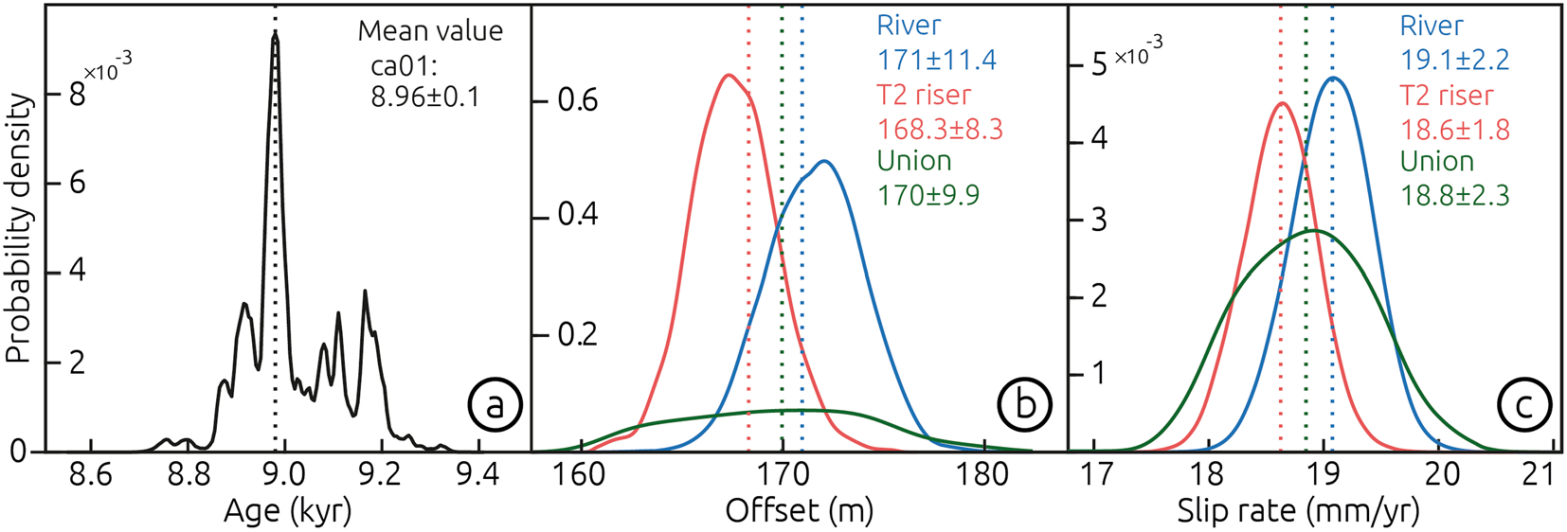
Slip rate of the Liquiñe Fault. (**a**) Probability distribution of the minimum abandonment age of T2 from OxCal modelling (Fig. S7). (**b**) Probability distribution for displacement estimates using T2, the bedrock river channel, and their union. C, Slip-rate distributions estimated using the method of Ref. 26 and MATLAB R2017a (www.mathworks.com).

We estimated the abandonment age of T2 using tephrochronology and radiocarbon dating (Fig. 4a). Details on the dating procedure may be found in the Methods section and in the Supplementary Materials (Text S2, Figs. S5–S7, Table S1 and S2). Our age model suggests that the two pumice layers interbedded within the T2 sediments correspond to the Neltume and Huilo pumices (Figs. 4 and S7), deposited during Plinian eruptions of the Mocho-Choshuenco volcano with inferred ages of 12.4–10.3 and 8.4–7.9 ka BP, respectively^30^. These are the largest post-glacial eruptions of this volcano with associated deposits that have been traced to the Liquiñe site in a regional survey^30^ (Fig. S5). A charcoal fragment from the paleosol horizon underlying the Huilo pumice yielded a modelled radiocarbon age of 9.0 ± 0.1 cal ka BP (Table S1 and Fig. 6a), supporting the tephrochronological results. By considering the stratigraphic context of this sample (paleosol at the top of the fluvial Unit B), we interpret this radiocarbon age to provides a minimum age for the abandonment of terrace T2. We propose that the abandonment of T2 occurred shortly before the formation of the paleosol horizon at 9.0 ka.

The nearly orthogonal relation between clast imbrications of T2 (Unit B) and T1 suggests that deposition of Unit B was associated with the westward-flowing Liquiñe River, likely before development of the pronounced deflection. The deflected river channel is associated with distinct structural features (Fig. 5a). At the southern shore, NNE-striking faults with subhorizontal slickensides affect granitic rocks across a 2-m-wide damage zone (Fig. 5b, c). Conglomerates of T2 are affected by N-S oriented transgranular fractures within a 2-m-wide shear zone of steeply-dipping aligned cobbles (Fig. 5d, e). The shear zone includes trans-granular fractures that affect both matrix and cobbles, and the dip distributions of fractures and of rotated cobbles are similar suggesting a common deformation mechanism (Fig. 5e). The river deflection is bounded to the west by a steep, up to 50-m-high, N–S oriented bedrock slope interpreted as a shutter ridge related to dextral fault slip (Figs. 3, 5a and S3c). This bedrock slope is locally affected by landslides. The development of a plunge pool at the southern part of the river inflection is related to the position of a 3-m-high knickpoint that has retreated ~ 50 m (Fig. S2a). We associate these structures affecting bedrock and fluvial conglomerates with a NNE-striking dextral fault parallel to the deflected river channel (Fig. 3).

### Holocene slip rate of the Liquiñe Fault

We estimate a Holocene slip rate for the Liquiñe Fault using two geomorphic markers offset by dextral fault slip: the bedrock channel and the riser above the terrace T2 surface (Fig. 3). Further details may be found in the Methods section and the Supplementary Materials (Figs. S4 and S8). The correlation of T2 across the fault and river is supported by similar elevation distributions as evident in topographic profiles (Fig. S4). By projecting the bedrock channel flanks and the T2 riser to the fault trace, we estimate mean offsets of 171.5 ± 8.2 m and 168.1 ± 8.7 m, respectively (Fig. 6b). These estimates are associated with extrapolation distances of 45 and 24 m, for western and eastern fault blocks of the bedrock channel, and 121 and 11 m for the T2 riser, respectively (Fig. 3a). The probability density functions of these two estimates have a 38% overlap and are equivalent within uncertainties, so we interpret that the accumulated fault slip postdates the abandonment and incision of T2. The ~ 3-m-high knickpoint east of the fault adjacent to the plunge pool (Fig. S2a) and ~ 5-m vertical displacement of T2 across a fault scarp (Fig. S3a and profile 6 in Fig. S4) suggest a vertical/horizontal displacement ratio of ~ 1/60 and thus predominant strike-slip motion along the Liquiñe Fault.

The abandonment age of terrace T2 provides a time constraint to estimate a slip rate for the Liquiñe Fault. The Neltume tuff has been associated with the largest eruption of the Mocho-Choshuenco volcano in post-glacial times^30^, which likely triggered widespread hillslope processes impacting sediment transport dynamics and possibly causing the onset of aggradation at the Liquiñe site and deposition of the T2 conglomerate. This phase of aggradation was superseded by soil formation implying a shift in the depositional environment likely by fluvial incision shortly before deposition of the Huilo tuff. The orthogonal directions of paleocurrents in T1 and T2, the linear and steep hillslope interpreted as a shutter ridge exposed on the western fault block between T1 and T2 (Fig. 3a), as well as the similar displacements recorded by the T2 riser and the present-day bedrock channel suggest that the abandonment of T2 predates fault displacement. Therefore, the abandonment age of T2 could be considered a maximum temporal marker to estimate a fault slip rate. Fluvial incision resetted the geomorphic strain marker of dextral slip along the Liquiñe Fault, and therefore by considering the 9.0 ± 0.1 ka age of T2 abandonment and 170.0 ± 11.6 m of mean offset, we estimate a slip rate of 18.8 ± 2.0 mm/year (95% confidence) for the Liquiñe Fault (Fig. 6); the determination of uncertainties follows the method of Ref.^31^. This rate should be considered a minimum estimate assuming that all the displacement was accumulated after T2 abandonment, as suggested by similarities in displacement of the T2 riser and bedrock channel; alternatively, it should be considered a maximum value if displacement was recorded prior to T2 abandonment, although this assessment is not supported by field observations.

## Discussion: millennial strain localization and slip behaviour

Our slip rate implies that ~ 82% of the trench-parallel component of oblique plate convergence has been accommodated along the Liquiñe Fault strand during the Holocene. The fault is apparently associated with scattered shallow microseismicity^10^ and located at about the centre of the ~ 30-km-wide LOFS. Therefore, at the Holocene timescale, most of the margin-parallel component of oblique convergence appears to have been accommodated within a narrow zone associated with a single fault strand, implying a high degree of slip partitioning and strain localization at millennial time scales.

We mapped the Liquiñe Fault as a continuous structure for a length of 32 km between the Las Pampas and Pirehueico lakes using deformed geomorphic features (Fig. 2). The northern end point of the Liquiñe Fault is marked by its intersection with the ~ 50-km-long, NW–SE oriented Villarica-Quetrupillán-Lanín volcanic alignment (Fig. 2), which has been associated with an underlying ~ 350-km-long sinistral fault^32^. To the south, the Liquiñe Fault loses its geomorphic expression across the Pirehueico Lake (Fig. 2). The length of the Liquiñe Fault and the 12-km thickness of the seismogenic layer^10^ suggest a fault area of 384 km^2^, which may generate a M_w_ 6.4 earthquake based on empirical relationships^33^. However, the fast Holocene slip rate would imply a recurrence interval of ~ 24 years for such earthquakes (i.e., Ref.^33^), which is unlikely based on the lack of historical earthquakes along this segment, and inconsistent with the ~ 2-kyr recurrence rate of LOFS-earthquakes at Aysén^16^. The Liquiñe Fault could be kinematically linked with the Los Guindos Fault^20^ that extends farther south with an end point at the Caulle Volcanic complex, which would imply a combined length of ~ 100-km and M_w_ ~ 7.0 earthquakes every ~ 150 year (i.e., Ref.^33^) (Fig. S9). However, such a scenario is not supported by the 500-year-long historical record. It is unlikely that large LOFS-earthquakes ruptured across volcanic complexes such as Caulle and Villarica-Quetrupillán-Lanín because of the lack of surface evidences and high heat flow associated with active volcanism (these are among the most active volcanoes in Chile). Assuming a recurrence rate of 500 year, which would exceed the length of historical records requires M_w_ ~ 7.3 earthquakes involving slip of > 9 m and a rupture length of ~ 220 km to account for the millennial slip rate (Fig. S9). The latter case is unlikely as the geomorphic signature of such large magnitudes of slip would be evident in the landscape.

An alternative explanation for the fast millennial slip rate and the lack of significant historical earthquakes along the Liquiñe Fault is that part of the slip may be accommodated aseismically by fault creep. This hypothesis could be tested further by a dedicated geodetic experiment. Aseismic fault creep may explain the lack of historical earthquakes in this part of the LOFS and the relatively moderate (M_w_ < 6.2) magnitude of earthquakes along the entire LOFS, but requires further field evidence. Widespread hydrothermal activity and volcanism along the LOFS may provide the mechanical conditions for fault creep. Large strike-slip fault systems such as the San Andreas and North Anatolian faults are associated with creeping segments similar in extent and slip rate to the Liquiñe Fault^34,35^. Given that moderate earthquakes have occurred along strike-slip faults associated with aseismic slip^36^, we cannot discard the possible occurrence of a M_w_ ~ 6 event along the Liquiñe Fault, and possibly also along other, similar fault strands of the LOFS that exhibit evidences of slip during the Holocene. Such a potential scenario needs to be incorporated into future evaluations of seismic hazards and associated risks.

## Methods

### Digital terrain model, geomorphic mapping, and fault-slip estimates

In order to map deformed geomorphic features at the densely-vegetated Liquiñe Site, we obtained a 50-cm-resolution Digital Terrain Model (DTM) using a terrestrial laser scanner and a differential GNSS system (for details see supplementary text S1 and Figs. S1 and S2). Mapping of deformed geomorphic features was carried out in the field using the DTM and satellite imagery, focused on fluvial terraces and fault-related features (scarps, shutter ridge, knickpoints) (Figs. 3, 5, S2, S3). For detailed mapping of the fluvial terraces, we used a Surface Classification Model (SCM), a semi-automatic algorithm to detect and map low-relief and gently-sloping areas commonly associated with terraces from a DTM^29^. We calculated a SCM map using slope and roughness thresholds of 35° and 0.8, respectively. In addition to mapping fluvial terraces, we levelled stratigraphic sections and measured the imbrication of fluvial clasts to infer paleocurrent directions using a clinometer in the field (Figs. 3b and S6). We surveyed 26 clasts in terrace T1 and 18 at each of the three sites in T2 (squares in Fig. 3b).

To estimate horizontal fault slip, we used two geomorphic markers, i.e., the modern thalweg and the riser of terrace T2. These offset markers were first identified and mapped at both sides of the fault in the field and then digitized using the DTM and satellite imagery as points (Fig. 2). In a second step, we estimated linear regressions from the mapped points and extrapolated them to the intersection with the Liquiñe Fault (Fig. 2). To estimate the associated uncertainties in the extrapolation, we obtained probability density functions from a bootstrap analysis of the linear regression coefficients using 10,000 samples. In order to further assess the role of epistemic uncertainties that may arise from the operators mapping geomorphic markers as well as the mapping resolution, we performed the same bootstrap analysis but in addition we randomly removed points from the digitized geomorphic markers. We removed random points progressively until diminishing the number of points by 50%. The results of this error simulation show that the mean offsets changed by only 0.2% and uncertainty estimates increase by 8% when 50% of the points are removed (Fig. S8).

### Tephrochronological correlations and age model

In order to assess the age of the deposits associated with terrace T2 and infer its abandonment age, we collected samples from the pumice layers in units A and C for tephrochronologic analysis (Table S2, Figs. 3c, e, S6), and a charcoal sample from the soil horizon at the top of unit B for radiocarbon dating (Table S1). For tephrochronology, we determined major element glass compositions with a JEOL JXA-8200 wavelength dispersive electron microprobe equipped with five spectrometers at the Institute of Geosciences, Potsdam University, and correlated this information with published geochemical characteristics of tephra deposits related to post-glacial eruptions of the Mocho-Choshuenco volcano, which reached the Liquiñe Site^30^ (Table S2, Figs. S5, S6 and S7). The results of the tephrochronologic correlations suggest that the Neltume and Huilo pumices are present in the deposits of T2. Table S2 and Figure S6 shows our results and the glass geochemical data of glasses from the Neltume and Huilo tephras. The tephrochronological results are validated by the radiocarbon age of the charcoal fragment (Table S1). To estimate the age of abandonment of T2, we combined our radiocarbon age with published ages of the Neltume and Huilo pumices (Table S1) in an OxCal model (using OxCal V4.3 by Ramsey 2017 (Ref. 35) and the ShCal13 curve^38^, obtaining a modelled age of 8.96 ± 0.1 cal ka BP for the charcoal fragment (Table S1, Fig. S7).

## Supplementary Information

Supplementary Information 1.

Supplementary Information 2.

## Data Availability

All data generated or analysed during this study are included in this published article (and its Supplementary Information files). Major element glass compositions for the sampled pumice layers are available at 4TU.ResearchData, according to FAIR Data standards (https://doi.org/10.4121/uuid:cbc3519c-d3e3-4dcb-82ed-cdfce0621434). The DTM is available at www.terracem.com, and the Raw Terrestrial Laser Scanner data and processed point-cloud data may be requested from the authors.
