# Supplementary material for: Fast Holocene slip and localized strain along the Liquiñe-Ofqui strike-slip fault system, Chile
Source: Sci Rep. 2021 Mar 16;11:5970. doi: 10.1038/s41598-021-85036-5 (PMC7966773; doi:10.1038/s41598-021-85036-5)
Supplement: Supplementary file 1 — Supplementary Information 1. [file 41598_2021_85036_MOESM1_ESM.pdf]

Supporting Information for

Fast Holocene slip and localized strain along the Liquiñe-Ofqui strike-slip fault system, Chile

Luis Astudillo-Sotomayor[1,2], Julius Jara-Muñoz[1,3], Daniel Melnick[1,4], Joaquín Cortés-Aranda[1,2], Andrés Tassara[1,2], Manfred R. Strecker[3]

[1]Millennium Nucleus The Seismic Cycle Along Subduction Zones, Valdivia, Chile.
[2]Departamento de Ciencias de la Tierra, Universidad de Concepción, Concepción, Chile.
[3]Institute of Geosciences, University of Potsdam, Potsdam, Germany.
[4]Instituto de Ciencias de la Tierra, TAQUACh, Universidad Austral de Chile, Valdivia, Chile.

Contents of this file

Text S1 to S2
Figures S1 to S9
Table S1

Additional Supporting Information (Files uploaded separately)

Table S2

Text S1

Processing of Terrestrial Laser Scanner data

The high-resolution topography of the Liquiñe site was obtained using a Riegl® LMS-Z260 long-range Terrestrial Laser Scanner (TLS). We scanned from four TLS locations (Fig. S1a), georeferenced using a Leica Viva dual-frequency GNSS system, and covering an area of 0.16 km^2. The raw data was processed and co-registered using the software RiSCAN PRO® obtaining a point cloud with a total of 104.2 million points and a mean point density of 99.6 pts/m^2. However, because of the topographic relief and relatively-dense vegetation, the point cloud density is not homogeneously-distributed throughout the area, which precluded the direct use of automated routines to filter-out vegetation and generate a bare-earth Digital Terrain Model (DTM). To overcome the heterogeneity in data distribution, we applied three processing steps to obtain a DTM (Fig. S1): 1) We first filtered the point cloud data using a standard LasTools® (www.rapidlasso.com) routine to remove first-returns from the forest canopy and noise, and keep last returns. 2) Then we used the ArcGIS LiDAR module to filter the point cloud manually by removing bumps, pits, and vegetation not correctly removed in the first step. However, the resulting LiDAR DTM still includes artefacts such as triangular facets and irregular surfaces mostly due to the effect of low-density returns from the river thalweg, which is partly covered by water precluding dense LiDAR returns (Fig. S1a). 3) finally, we applied a last processing step based on the hydrologic enforcement technique (Mäkinen et al., 2019; Parrot & Ramírez Núñez, 2016), which consists of masking the water surface and using this mask to correct the concealed areas using a constant elevation. We used Digital Globe satellite image (0.1 m-resolution) to map the water surface in the valley at the border of the surveyed area (Fig. S1c). This correction produced sharp edges in the topography at the borders of the water mask. We corroborated the presence of these topographic features in the field where the river incised into the granitic bedrock carving a canyon (Fig. S2a and S2b).

Text S2

Age Model Script

The OxCal 4.3.2 script used to calibrate and model radiocarbon ages of the Neltume and Huilo pumices, and to constrain the timing of terrace T2 abandonment is shown below. Boundary (unit name), R_date (lab code/sample name, conventional radiocarbon age, 1 sigma value). Ordered from oldest (top) to youngest (base). The resulting age model is shown in Figure S5 and Table S1.

- Options()
  - Curve="SHCal13.14c";
  - BCAD=FALSE;
- Plot()
  - Outlier_Model("General",T(5),U(0.4),"t");
  - Sequence("liquine")
    - Boundary("start");
    - Phase("below MC4")
      - R_Date("suerc-51918", 10349, 43)
        - Outlier(0.05);
      - R_Date("200892-02", 10660, 140)
        - Outlier(0.05);
    - Boundary("MC4");
    - Boundary("Conglomerates");
    - Phase("below MC9")
      - R_Date("020300-4a", 8160, 100)
        - Outlier(0.05);
      - R_Date("suerc-51921", 7627, 41)
        - Outlier(0.05);
      - R_Date("ca01", 8114, 46)
        - Outlier(0.05);
    - Boundary("MC9");
    - Phase("above MC9")
      - R_Date("beta-156226", 7260, 70)
        - Outlier(0.05);
      - R_Date("Ch-7", 6710, 90)
        - Outlier(0.05);
    - Boundary("end");

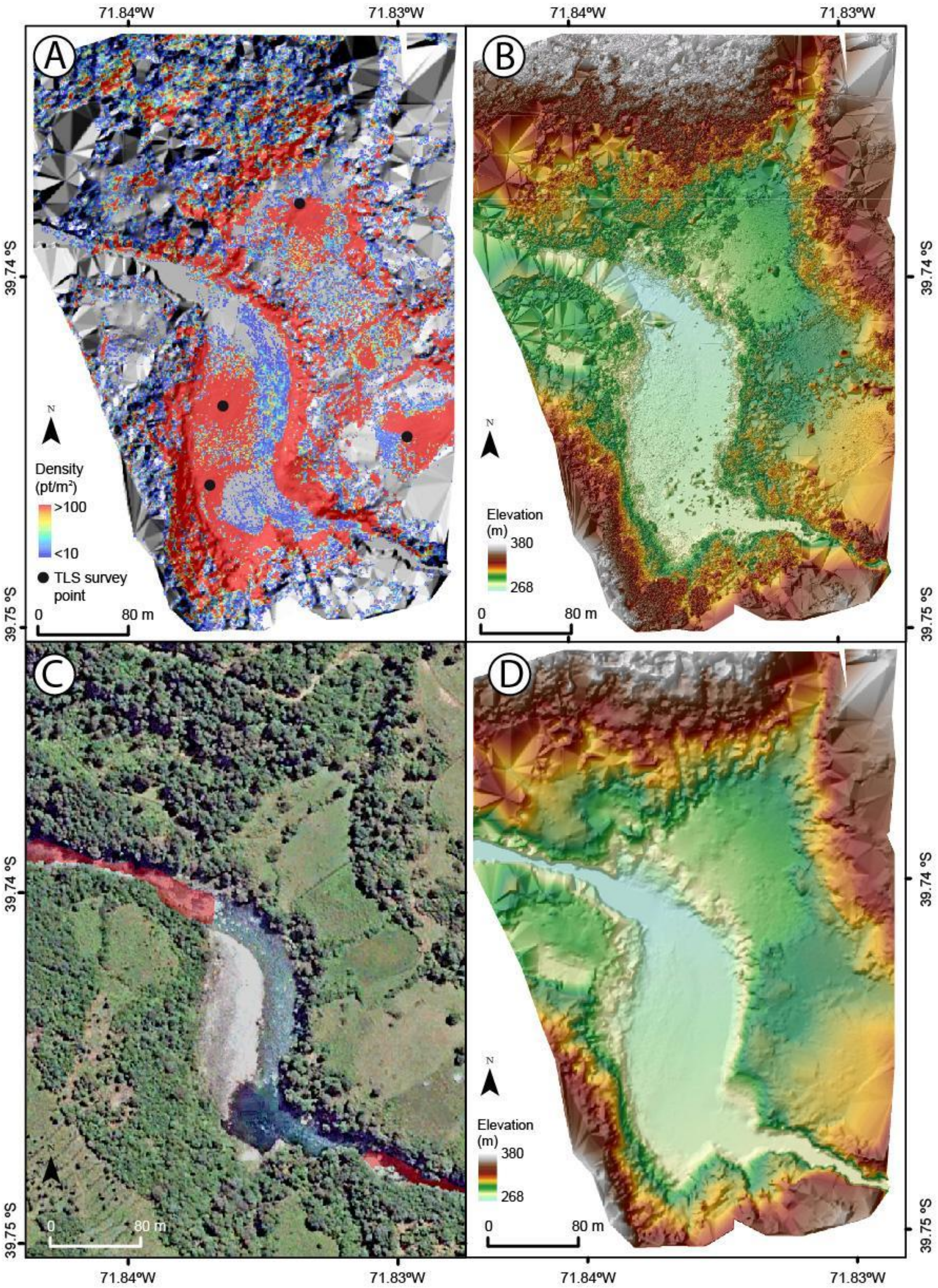

Figure S1. Terrestrial Laster Scanner (TLS) data processing steps, maps made using Qgis 3.10 (www.qgis.org). a) Point cloud density and scanner survey positions. b) Digital Surface Model including all the point cloud returns. c) Water mask digitized from Digital Globe satellite imagery used for hydrologic enforcement. d) Filtered Digital Terrain Model using LAStools (www.rapidlasso.com) and hydrologic enforcement (see text S1).

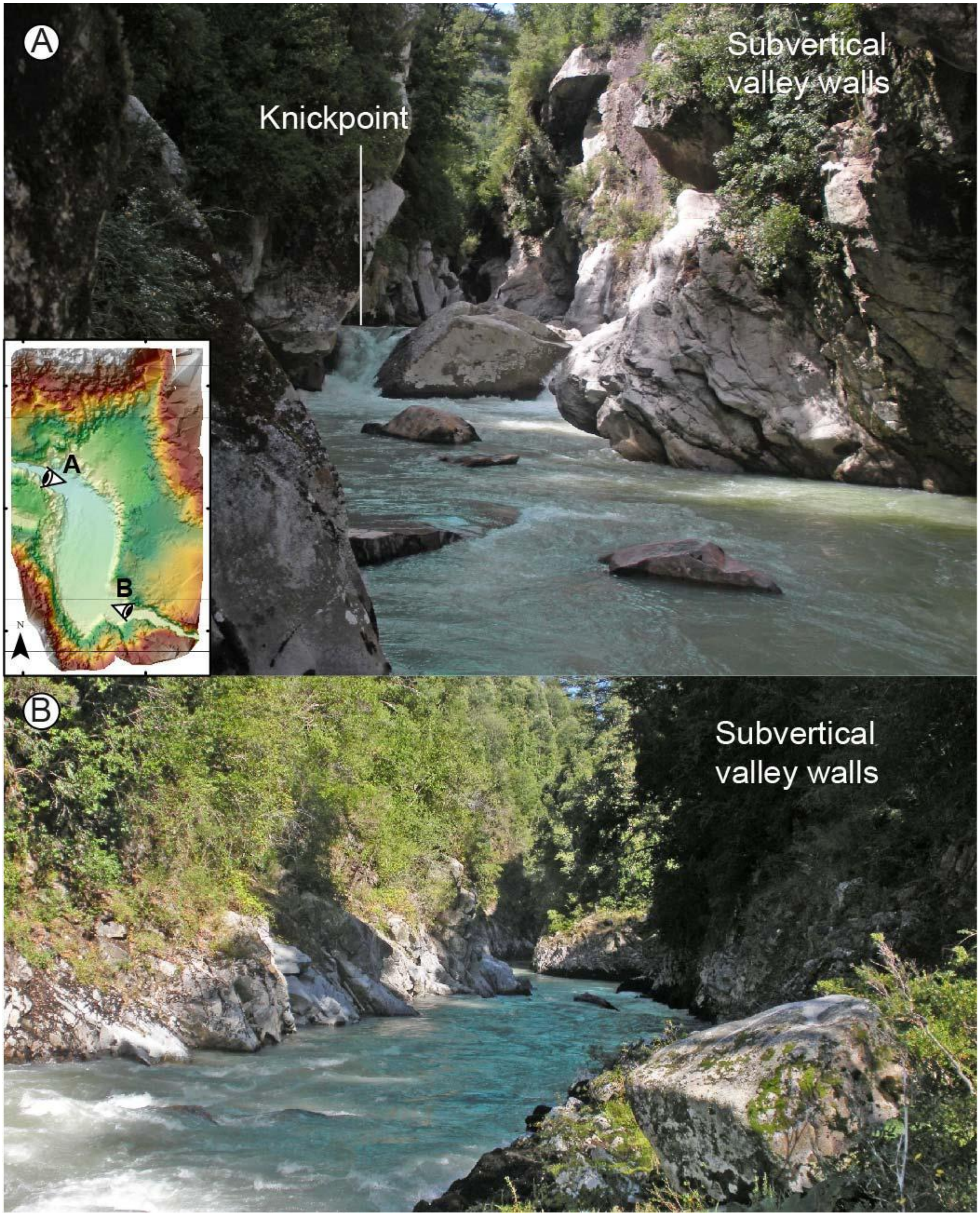

Figure S2. Channel incision along the Liquiñe River. a) View of the bedrock canyon at the northwestern part of the Liquiñe Site. Inset shows shaded-relief map with location of the field views. b) View of the canyon at the southeastern part.

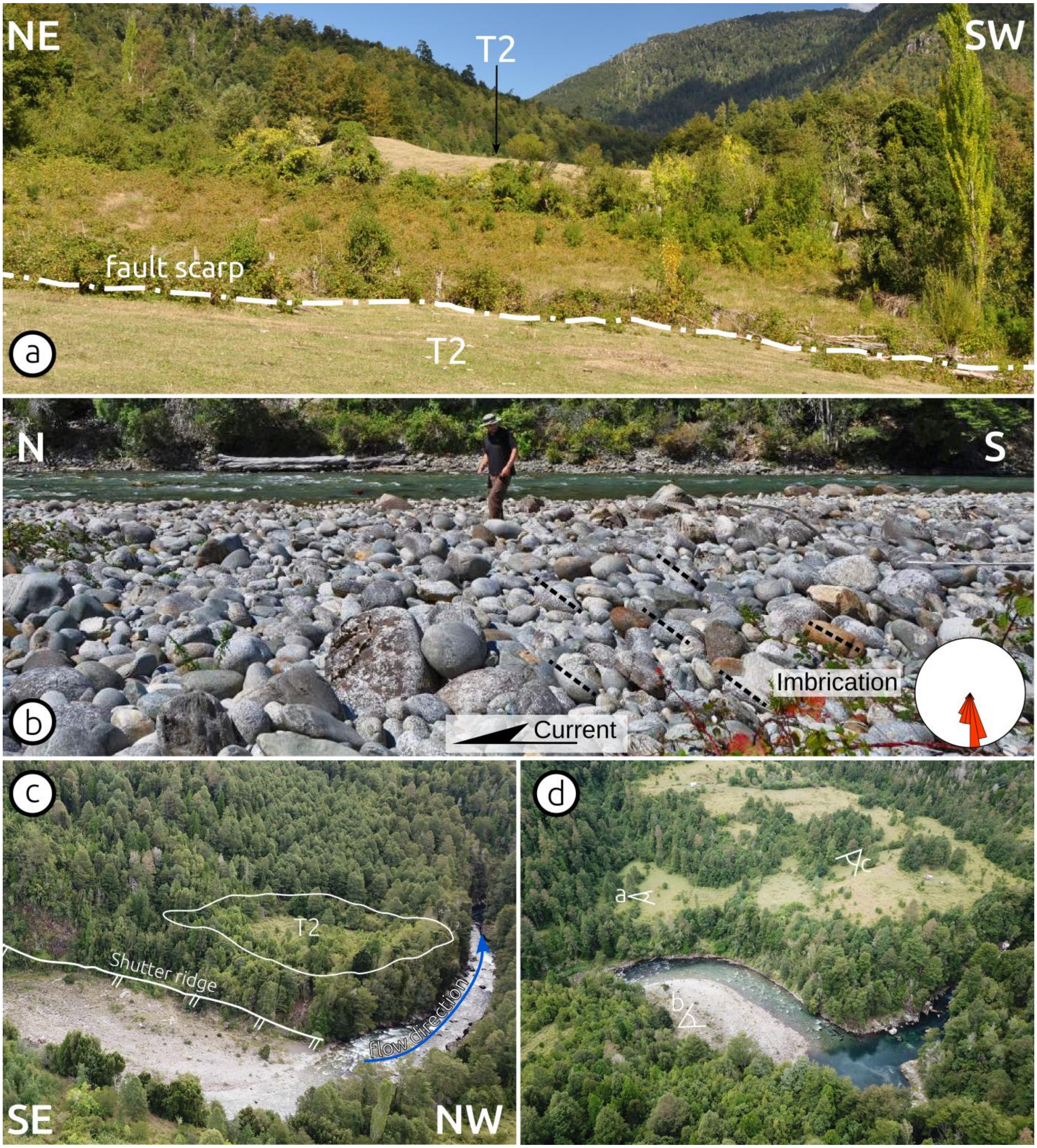

Figure S3. Geomorphic evidence of fault activity at the Liquiñe Site. a) Fault scarp affecting terrace T2 surface, north of the Liquiñe River. b) View of the modern floodplain of the Liquiñe River showing clast imbrications. Rose plot in inset shows measurement distribution. c) Oblique aerial photo showing the shutter ridge and extent of terrace T2 along the western block of the Liquiñe Fault. d) Oblique aerial photo of the Liquiñe Site showing location and view direction of photos in panels a, b, and c.

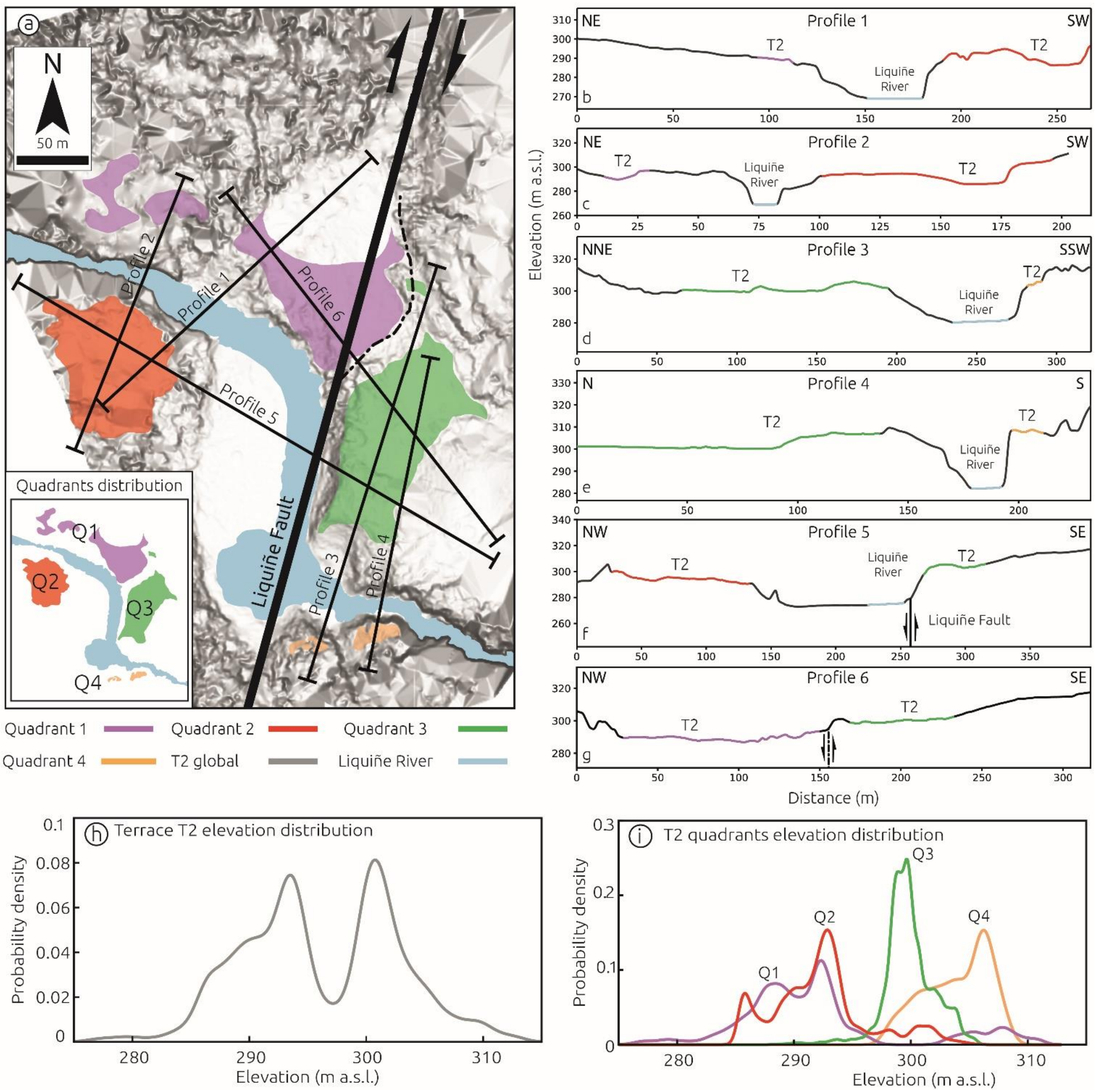

Figure S4. Correlation of T2 across the Liquiñe site. a) Map showing T2 quadrants and location of the topographic profiles. a-g) topographic profiles showing the distribution of T2 across the Liquiñe River and mapped structures. Coloured line segments correspond to the different segments depicted in A. h) Elevation distribution for the entire T2 terrace. Bimodal distribution associated with the ~5-m-high fault scarp that disrupts the terrace. i) elevation distribution of T2 quadrants. Quadrant distributions are consistent with their location with respect to the fault scarp.

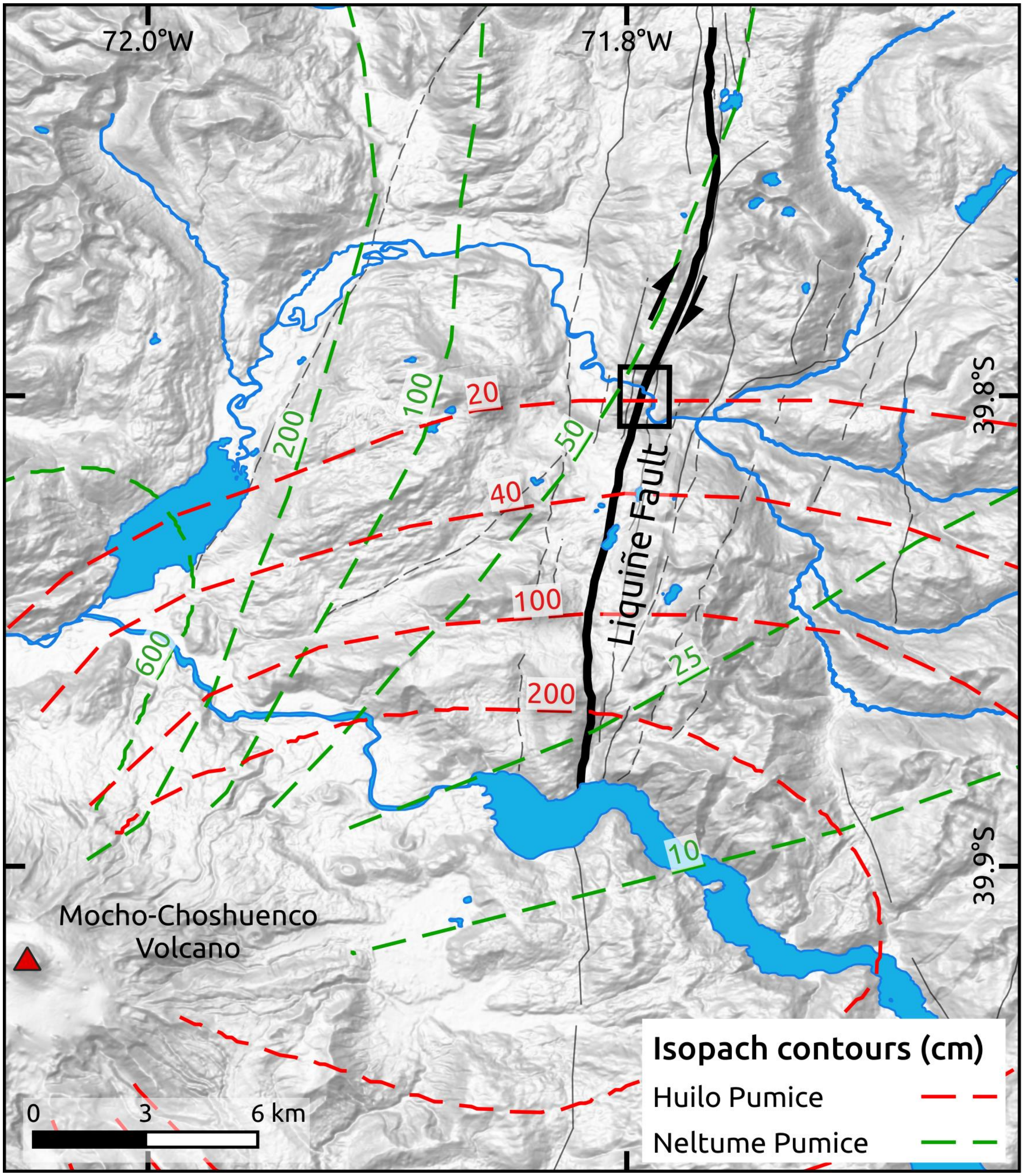

Figure S5. Shaded-relief map with isopach contours for the Neltume and Huilo pumices (Rawson et al., 2015), and mayor faults in the Liquiñe region.

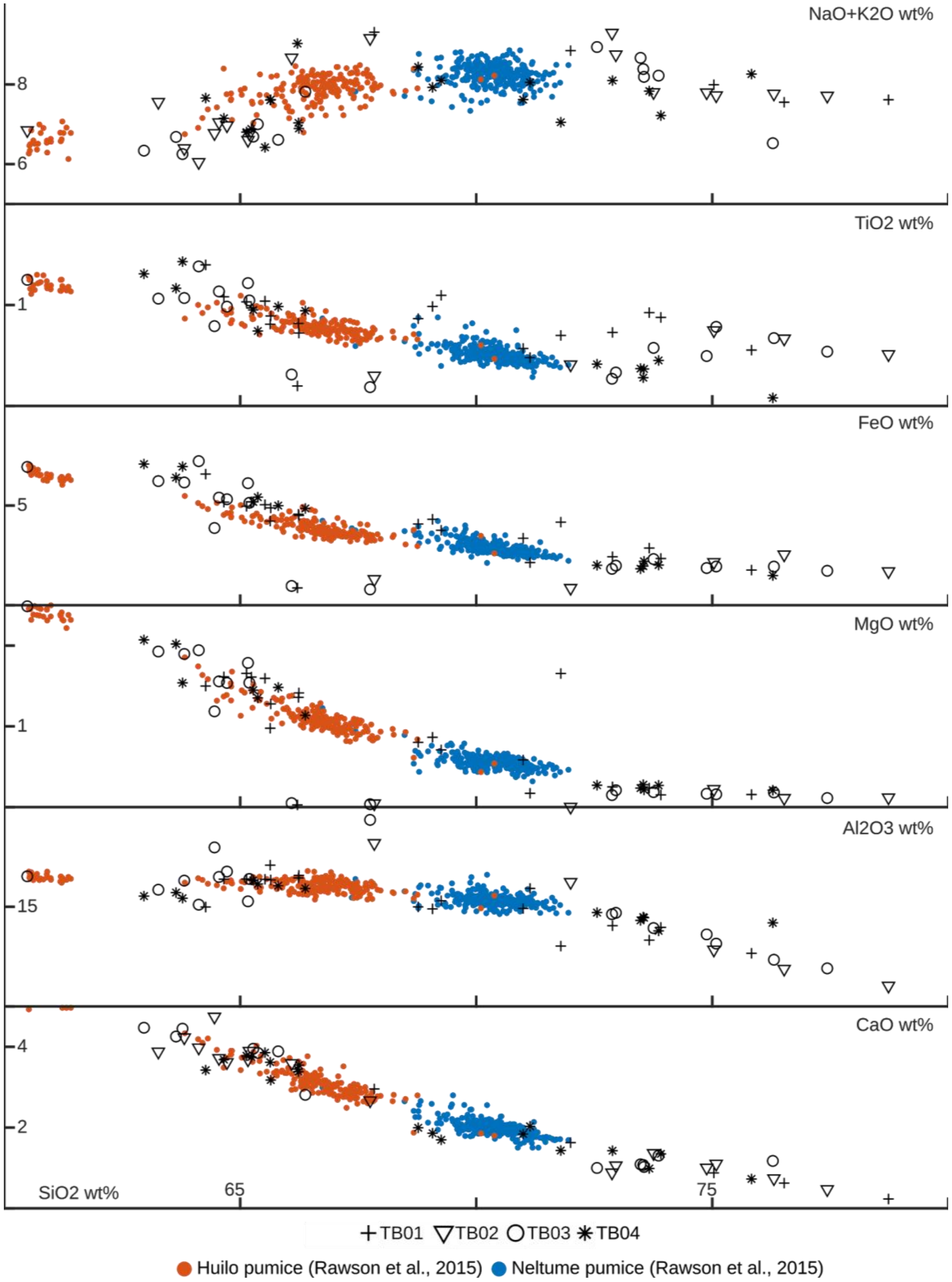

Figure S6. Harker diagrams for the Neltume and Huilo pumices from Rawson et al. (2015) and our samples from both pumices collected at the Liquiñe Site. Results from analyses are shown in Table S2.

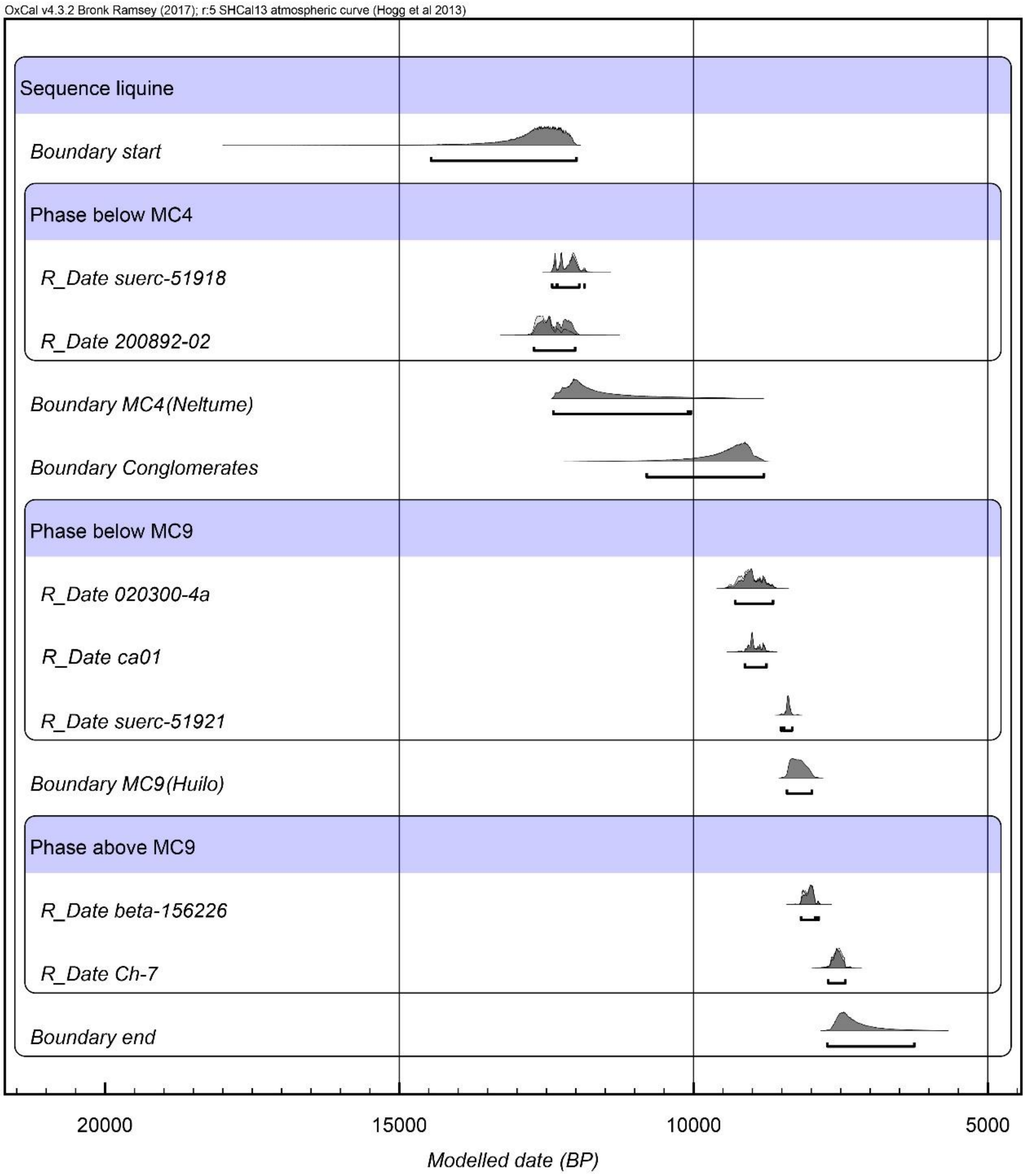

Figure S7. OxCal v 4.3 age model plot. Input ages in Table S1, model script in Text S2. Figure made using OxCal v 4.3 (https://c14.arch.ox.ac.uk/oxcal.html). MC9 and MC4 are Mocho-Choshuenco Volcano eruptions 9 and 4 (Rawson et al., 2015).

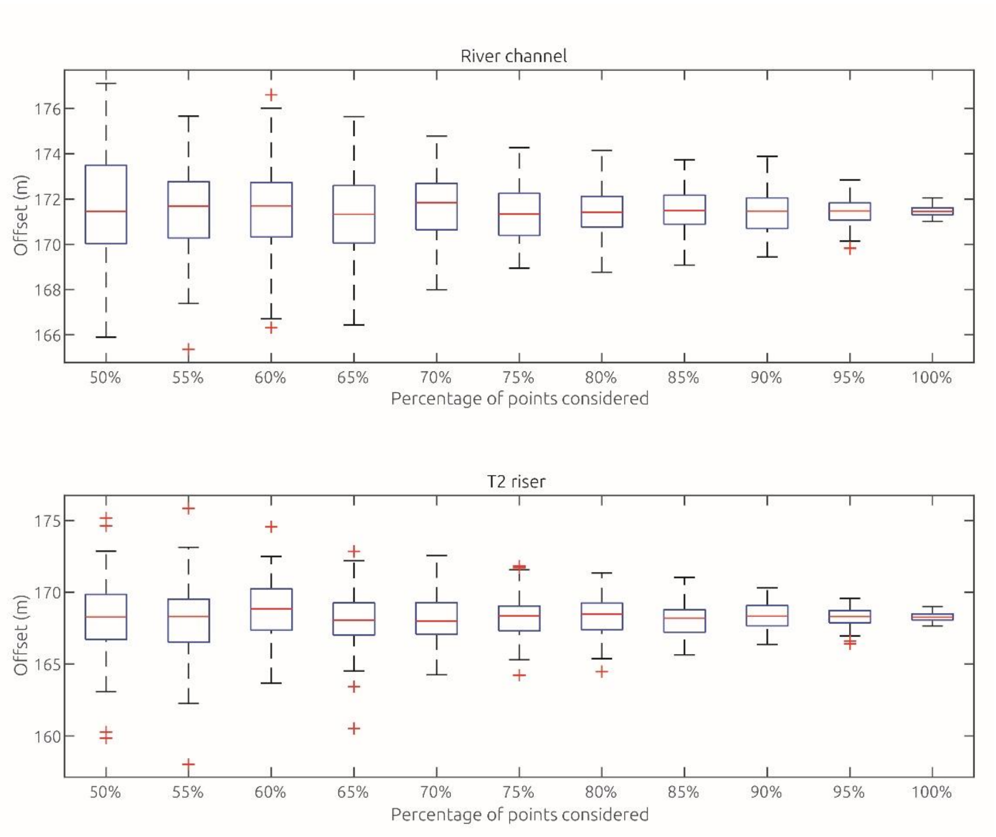

Figure S8. Error simulation results of the two offset markers used to estimate fault slip. See Methods and text for details. a) Box-and-whisker plots showing simulation results for river channel. Red line shows mean values. b) Box-and-whisker plots showing results for the T2 riser.

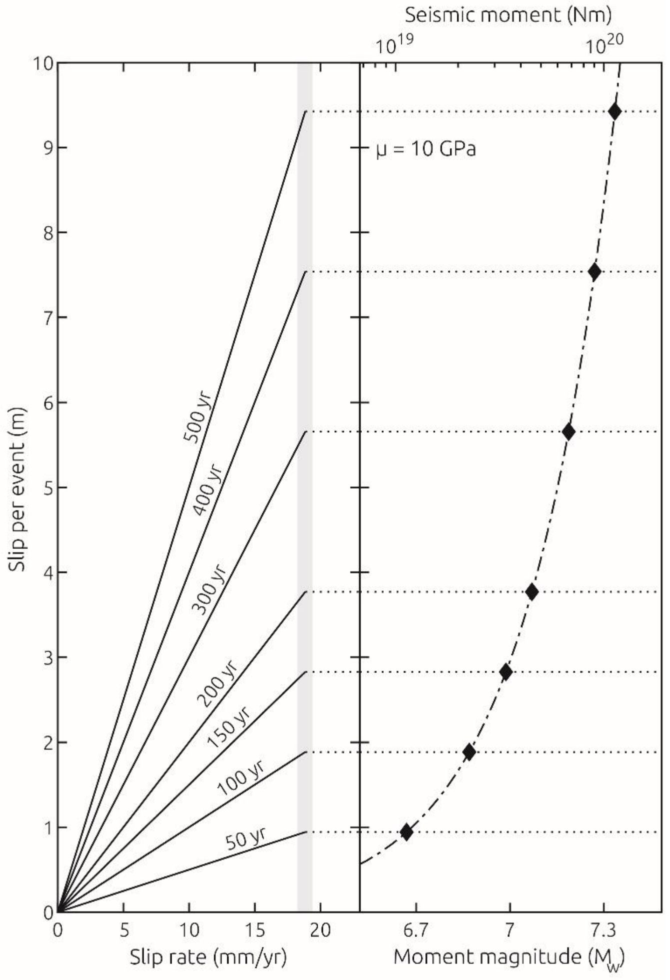

Figure S9. Earthquake scenarios for the Liquiñe Fault. a) slip per event assuming different recurrence times as a function of millennial slip rate. Grey line shows Holocene slip rate estimated at the Liquiñe site. b) Seismic moment and moment magnitude estimates for each slip scenario considering a rigidity modulus (μ) of 10 GPa (Heap et al., 2020) and 12 km depth seismogenic width (Sielfeld et al., 2019) following (Thingbaijam et al., 2017).

Unit/Sample	Stratigraphic position	Uncalibrated yrs BP	Unmodelled (BP)						Modelled (BP)						Reference	Method	Sample Material dated
			from	to	%	μ	σ	m	from	to	%	μ	σ	m			
SUERC-51918	bellow MC4	10349 ± 43	12399	11838	95.4	12126	143	12090	12403	11851	95.4	12153	140	12128	3	AMS	Carbonised twigs in soil at base of tephra deposit
200892-02	bellow MC4	10660 ± 140	12744	12056	95.4	12485	188	12572	12709	12014	95.4	12361	209	12381	1.5	Radiometric	
MC4	-	-	-	-	-	-	-	-	12387	10057	95.4	11586	660	11821		-	
Conglomerates	-	-	-	-	-	-	-	-	10840	8803	95.3	9540	559	9366		-	
020300-4a	bellow MC9	8160 ± 100	9400	8658	95.4	9048	172	9060	9291	8649	95.4	9001	168	9019	3	Radiometric	Charcoal
suerc-51921	bellow MC9	7627 ± 41	8514	8320	95.4	8392	39	8392	8515	8326	95.4	8397	39	8395	3	AMS	top 3cm of an organic rich soil with small pieces of charcoal directly below tephra layer.
la18liqca01	bellow MC9	8114 ± 46	9133	8761	95.4	8966	105	8997	9130	8761	95.4	8954	105	8989	2	AMS	Charcoal
MC9	-	-	-	-	-	-	-	-	8414	7988	95.4	8218	118	8226		Radiometric	
beta-156226	above MC9	7260 ± 70	8183	7879	95.4	8050	76	8042	8175	7875	95.4	8033	73	8024	3	Radiometric	Carbonised small branches
Ch-7	above MC9	6710 ± 90	7687	7420	95.4	7543	73	7541	7711	7422	95.4	7562	76	7561	1.4	Radiometric	

Table S1. Radiocarbon ages of the post-glacial eruptions from the Mocho-Choshuenco volcano. [1] (Moreno & Lara, 2007), [2] this work, [3] (Rawson et al., 2015), [4] (Echegaray, 2004) , [5] (Moreno et al., 1994). See Text S2 and Figure S5 for OxCal V4.3 model script and results.

Table S2 (Uploaded separately). Major oxides composition of samples from the Neltume and Huilo pumices collected at the Liquiñe Site (Fig. S4).

Supplementary References

Echegaray, J. (2004). *Evolución geológica y geoquímica del Centro Volcánico Mocho-Choshuenco, Andes del Sur*. Master Thesis, Universidad de Chile, Chile.

Mäkinen, V., Oksanen, J., & Sarjakoski, T. (2019). Automatic determination of stream networks from DEMs by using road network data to locate culverts. *International Journal of Geographical Information Science: IJGIS*, *33*(2), 291–313.

Moreno, H., Clavero, J., & Lara, L. (1994). Actividad explosiva postglacial del volcán Villarrica, Andes del Sur (39 25'S). *Congreso Geológico Chileno*, 329–333.

Moreno, H., & Lara, L. (2007). Geología del complejo volcánico Mocho-Choshuenco, Región de Los Ríos. Servicio Nacional de Geología y Minería. *Carta Geológica de Chile, Serie Geología Básica*, *107*, 27.

Parrot, J. F., & Ramírez Núñez, C. (2016). Artefactos y correcciones a los Modelos Digitales de Terreno provenientes del LiDAR. *Investigaciones Geográficas*, *2016*(90), 28–39.

Heap, M. J., Villeneuve, M., Albino, F., Farquharson, J. I., Brothelande, E., Amelung, F., Got, J., & Baud, P. (2020). Towards more realistic values of elastic moduli for volcano modelling. *Journal of Volcanology and Geothermal Research*, *390*, 106684. https://doi.org/10.1016/j.jvolgeores.2019.106684

Rawson, H., Naranjo, J. A., Smith, V. C., Fontijn, K., Pyle, D. M., Mather, T. A., & Moreno, H. (2015). The frequency and magnitude of post-glacial explosive eruptions at Volcán Mocho-Choshuenco, southern Chile. *Journal of Volcanology and Geothermal Research*, *299*, 103–129. https://doi.org/10.1016/j.jvolgeores.2015.04.003

Sielfeld, G., Lange, D., & Cembrano, J. (2019). Intra-Arc Crustal Seismicity: Seismotectonic Implications for the Southern Andes Volcanic Zone, Chile. *Tectonics*, *38*(2), 552–578. https://doi.org/10.1029/2018TC004985

Thingbaijam, K. K. S., Mai, P. M., & Goda, K. (2017). New empirical earthquake source-scaling laws. *Bulletin of the Seismological Society of America*, *107*(5), 2225–2246. https://doi.org/10.1785/0120170017
