# Supplementary material for: Fast Holocene slip and localized strain along the Liquiñe-Ofqui strike-slip fault system, Chile
Source: Sci Rep. 2021 Mar 16;11:5970. doi: 10.1038/s41598-021-85036-5 (PMC7966773; doi:10.1038/s41598-021-85036-5)
Supplement: Supplementary file 2 — Supplementary Information 2. [file 41598_2021_85036_MOESM2_ESM.pdf]

TABLE S2. GLASS MICROPROBE ANALYSES (wt%)

Sample number	Na2O	Al2O3	TiO2	FeO	K2O	SiO2	MgO	Cr2O3	MnO	CaO	Total	Location*
TB01												
70	0.09	0.65	0.32	17.68	0.03	54.25	25.04	0.00	1.26	1.36	100.69	4-1
71	6.09	27.73	0.00	0.47	0.20	58.28	0.05	0.00	0.00	8.63	101.44	4-1b
72	0.32	1.95	0.64	9.08	0.00	52.51	15.02	0.00	0.55	20.24	100.30	4-1c
73	6.38	27.64	0.19	0.57	0.14	56.74	0.05	0.00	0.00	8.95	100.67	4-1d
74	5.46	28.25	0.00	0.41	0.11	54.84	0.06	0.07	0.00	10.50	99.69	4-1e
75	7.10	26.37	0.08	0.45	0.23	59.26	0.04	0.00	0.00	7.85	101.38	4-1f
76	0.09	1.65	0.36	15.09	0.00	54.24	27.52	0.05	0.44	1.63	101.07	4-1g
80	5.59	28.10	0.00	0.96	0.35	56.47	0.10	0.00	0.02	10.56	102.15	4-1h
81	4.49	12.61	0.73	2.12	3.36	73.68	0.22	0.00	0.12	0.86	98.20	4-1i
83	3.69	11.57	0.65	2.47	3.66	74.48	0.11	0.00	0.09	0.61	97.33	4-1k
77	0.00	1.15	0.26	17.93	0.00	54.38	25.09	0.02	0.99	1.76	101.59	4-2
78	6.69	16.30	0.41	0.87	2.19	72.30	0.00	0.00	0.02	1.63	100.41	4-2c
79	3.49	10.86	0.50	1.67	4.01	77.50	0.12	0.02	0.06	0.23	98.45	4-2d
82	7.23	18.08	0.30	1.30	2.02	67.43	0.04	0.05	0.06	2.94	99.44	4-lj
TB02												
1	5.13	16.35	0.96	5.19	1.67	63.11	1.50	0.00	0.09	3.53	97.52	5-1
10	5.52	18.09	0.80	3.90	1.30	64.86	1.19	0.00	0.20	4.77	100.63	5-10
11	5.00	16.34	1.24	6.86	1.77	59.80	2.46	0.00	0.19	5.20	98.85	5-11
12	4.86	14.69	0.33	1.98	3.88	72.91	0.21	0.00	0.01	1.06	99.94	5-12
13	5.16	14.41	0.27	1.80	4.00	71.81	0.15	0.04	0.08	0.87	98.57	5-13
41	0.00	0.01	0.00	0.00	0.02	100.07	0.03	0.00	0.00	0.00	100.13	5-13b
42	0.00	24.68	0.05	0.14	0.03	43.94	0.01	0.00	0.02	27.49	96.37	5-13c
58	4.83	12.29	0.67	1.93	2.90	75.99	0.18	0.00	0.05	0.74	99.59	5-1b
59	4.94	13.64	0.50	1.87	2.87	75.03	0.17	0.04	0.18	1.01	100.23	5-1c
2	7.02	19.98	0.31	0.95	1.50	64.96	0.05	0.02	0.00	3.53	98.31	5-2
3	4.97	15.91	1.01	4.99	1.60	63.28	1.50	0.00	0.02	3.78	97.07	5-3
60	4.50	13.06	0.78	1.93	3.17	74.57	0.16	0.00	0.06	1.09	99.31	5-3b
4	6.82	25.08	0.23	1.08	0.40	57.07	0.15	0.05	0.04	7.15	98.07	5-4
5	5.19	15.77	1.06	6.19	2.32	62.91	1.92	0.05	0.23	3.86	99.49	5-5
61	4.01	11.78	0.53	1.71	3.62	76.60	0.11	0.00	0.10	0.47	98.92	5-5b
6	4.06	14.55	1.33	6.96	1.77	61.76	1.87	0.00	0.19	3.83	96.32	5-6
62	4.91	13.86	0.57	2.30	2.85	73.39	0.19	0.03	0.07	1.36	99.52	5-6b
7	5.54	26.17	0.02	1.12	0.21	57.62	0.16	0.00	0.00	9.10	99.94	5-7
8	4.64	15.72	1.03	5.94	1.52	61.49	1.83	0.02	0.12	4.08	96.37	5-8
63	4.87	15.14	1.21	6.07	1.67	64.59	1.77	0.00	0.15	3.65	99.12	5-8b
64	7.67	19.45	0.19	0.80	1.53	68.07	0.04	0.00	0.04	2.68	100.46	5-8c
9	5.04	15.80	1.09	5.17	1.71	61.81	1.49	0.01	0.09	3.56	95.76	5-9
TB03												
14	5.06	29.46	0.16	0.59	0.12	54.28	0.08	0.00	0.00	11.60	101.35	6-1
23	4.46	15.59	1.16	6.35	2.17	63.13	2.00	0.05	0.12	4.22	99.24	6-10
24	5.43	13.89	0.45	2.03	2.85	74.39	0.27	0.00	0.09	1.31	100.72	6-11
15	5.23	16.03	0.74	5.38	1.72	64.92	1.34	0.00	0.12	3.82	99.31	6-2
16	4.92	16.08	0.99	5.00	1.70	65.88	1.48	0.08	0.17	3.89	100.19	6-3
17	4.53	14.39	0.28	1.95	3.80	73.04	0.28	0.02	0.00	1.07	99.34	6-4
43	4.37	14.41	0.37	2.20	3.80	73.36	0.21	0.00	0.00	1.02	99.74	6-4b
44	4.38	13.66	0.35	1.74	3.88	70.08	0.23	0.00	0.01	1.04	95.37	6-4c
18	4.88	16.13	0.94	5.12	1.73	64.51	1.43	0.00	0.18	3.91	98.83	6-5
19	3.39	13.80	0.08	1.45	2.96	74.17	0.20	0.02	0.05	1.14	97.25	6-6
20	5.04	15.80	0.94	4.82	2.72	65.88	1.13	0.00	0.14	2.79	99.24	6-7
21	5.21	14.71	0.41	2.01	3.73	72.55	0.27	0.06	0.11	1.00	100.04	6-8
22	4.33	30.81	0.00	0.87	0.09	53.50	0.05	0.00	0.02	12.83	102.49	6-9
39	4.74	15.60	1.31	7.11	1.63	63.20	2.08	0.03	0.23	4.49	100.41	6-9b
40	4.56	15.31	1.42	6.90	1.64	63.27	1.53	0.06	0.17	4.41	99.26	6-9c
TB04												
25	4.60	12.66	0.55	1.76	3.66	75.83	0.16	0.05	0.05	0.73	100.04	7-1
33	5.43	16.51	1.09	5.21	1.78	65.14	1.63	0.00	0.26	3.71	100.75	7-10
47	5.03	14.82	0.57	3.34	2.54	70.52	0.58	0.00	0.12	1.83	99.34	7-10b
34	4.76	15.52	1.11	3.81	3.45	70.22	0.72	0.03	0.09	1.72	101.41	7-11
45	4.95	15.05	0.87	4.10	3.52	69.03	0.80	0.01	0.06	2.00	100.38	7-11b
46	4.51	14.89	0.98	4.31	3.41	69.02	0.86	0.01	0.08	1.86	99.93	7-11c
35	5.21	30.39	0.07	0.33	0.27	55.12	0.04	0.00	0.00	11.43	102.87	7-12
52	4.77	16.15	1.02	4.97	1.56	64.60	1.57	0.00	0.14	3.80	98.59	7-12b
53	2.52	33.91	0.04	0.84	0.04	48.78	0.07	0.01	0.00	16.41	102.62	7-12c
26	5.26	16.46	0.82	4.57	1.79	66.27	1.36	0.07	0.15	3.39	100.13	7-2
48	5.50	15.84	0.48	2.13	2.51	70.77	0.17	0.00	0.07	2.02	99.48	7-2b

54	5.12	16.48	0.72	4.48	1.73	65.86	1.41	0.02	0.10	3.53	99.44	7-2b
49	7.08	25.48	0.07	0.70	0.33	60.64	0.08	0.08	0.00	7.07	101.52	7-2c
55	5.15	14.09	0.73	2.44	2.97	73.07	0.25	0.00	0.13	1.43	100.25	7-2c
50	4.45	13.87	0.87	2.33	2.72	73.41	0.15	0.00	0.19	1.34	99.32	7-2d
56	4.38	13.03	0.70	4.17	2.67	71.81	1.65	0.00	0.18	1.43	100.03	7-2d
57	4.62	13.39	0.93	2.88	3.26	74.07	0.24	0.00	0.17	0.99	100.55	7-2e
27	0.35	2.19	0.69	9.49	0.00	52.54	15.50	0.01	0.39	20.51	101.67	7-3
65	7.89	20.44	0.20	0.88	1.24	66.94	0.03	0.00	0.00	3.48	101.10	7-3b
66	6.35	28.37	0.03	0.50	0.15	57.49	0.05	0.02	0.00	9.63	102.59	7-3c
28	5.32	16.31	0.97	5.18	1.56	65.12	1.61	0.00	0.02	3.74	99.82	7-5
29	5.07	15.09	1.41	6.63	2.65	64.74	1.51	0.00	0.19	3.45	100.73	7-6
67	7.60	25.98	0.08	0.43	0.44	60.75	0.08	0.01	0.06	7.00	102.42	7-6b
30	5.74	29.20	0.01	0.08	0.09	56.67	0.02	0.00	0.02	10.38	102.22	7-7
68	6.74	26.92	0.02	0.63	0.28	58.82	0.03	0.01	0.00	7.99	101.43	7-7b
31	5.15	16.37	1.03	4.96	1.65	65.03	1.65	0.00	0.24	3.77	99.84	7-8
32	5.99	17.15	0.81	4.24	1.64	65.87	0.98	0.00	0.05	3.63	100.35	7-9
51	5.75	16.29	0.89	4.86	1.85	65.44	1.27	0.00	0.16	3.17	99.68	7-9b
69	6.09	28.28	0.10	0.46	0.15	57.65	0.09	0.01	0.03	9.72	102.58	7-9b
Minimum	0.00	0.01	0.00	0.00	0.00	43.94	0.00	0.00	0.00	0.00	95.37	
Maximum	7.89	33.91	1.42	17.93	4.01	100.07	27.52	0.08	1.26	27.49	102.87	
Average	4.79	17.03	0.58	3.55	1.79	65.24	1.97	0.01	0.12	4.75	99.84	
Sigma	1.75	7.21	0.42	3.52	1.30	8.44	5.33	0.02	0.19	5.00	1.62	
No. of data	80											
